# Activation of MyD88 Signaling upon Staphylococcal Enterotoxin Binding to MHC Class II Molecules

**DOI:** 10.1371/journal.pone.0015985

**Published:** 2011-01-20

**Authors:** Teri L. Kissner, Gordon Ruthel, Shahabuddin Alam, Robert G. Ulrich, Stefan Fernandez, Kamal U. Saikh

**Affiliations:** Department of Immunology, Army Medical Research Institute of Infectious Diseases, Frederick, Maryland, United States of America; Centre de Recherche Public de la Santé (CRP-Santé), Luxembourg

## Abstract

Ligands binding to Toll-like receptor (TLR), interleukin 1 receptor (IL-1R), or IFN-γR1 are known to trigger MyD88-mediated signaling, which activates pro-inflammatory cytokine responses. Recently we reported that staphylococcal enterotoxins (SEA or SEB), which bind to MHC class II molecules on APCs and cross link T cell receptors, activate MyD88- mediated pro-inflammatory cytokine responses. We also reported that MyD88^−/−^ mice were resistant to SE- induced toxic shock and had reduced levels of serum cytokines. In this study, we investigated whether MHC class II- SE interaction by itself is sufficient to activate MyD88 in MHC class II^+^ cells and induce downstream pro-inflammatory signaling and production of cytokines such as TNF-α and IL-1β. Here we report that human monocytes treated with SEA, SEB, or anti-MHC class II monoclonal antibodies up regulated MyD88 expression, induced activation of NF-kB, and increased expression of IL-1R1 accessory protein, TNF-α and IL-1β. MyD88 immunoprecipitated from cell extracts after SEB stimulation showed a greater proportion of MyD88 phosphorylation compared to unstimulated cells indicating that MyD88 was a component of intracellular signaling. MyD88 downstream proteins such as IRAK4 and TRAF6 were also up regulated in monocytes after SEB stimulation. In addition to monocytes, primary B cells up regulated MyD88 in response to SEA or SEB stimulation. Importantly, in contrast to primary B cells, MHC class II deficient T2 cells had no change of MyD88 after SEA or SEB stimulation, whereas MHC class II-independent activation of MyD88 was elicited by CpG or LPS. Collectively, these results demonstrate that MHC class II utilizes a MyD88-mediated signaling mechanism when in contact with ligands such as SEs to induce pro-inflammatory cytokines.

## Introduction

In addition to their role as restricting elements in antigen presentation, major histocompatibility complex (MHC) class II molecules can trigger intracellular signals after ligand binding in many cell types. Staphylococcal enterotoxins (SE), a group of MHC class II binding proteins secreted by *Staphylococcus aureus* [1]–[5], and monoclonal antibodies (mAb) against MHC-class II molecules induce activation of tyrosine kinases, turnover of membrane phosphoinositol, and expression of inflammatory cytokine genes in monocytic cells [6]–[8]. Ligation of MHC class II molecules in B cells also triggers intracellular Ca^2+^ mobilization and homotypic adhesion [9], [10]. MHC class II dependent induction of IL-1 gene expression and B-cell aggregation are dependent on the activation of tyrosine kinases and protein tyrosine kinase C [6], [8], [11], [12]. Initiation of the principal signaling pathway of MHC class II molecules in human B lymphocytes requires the HLA-DRβ cytoplasmic tail [13].

MyD88 is an essential anchor protein that integrates and transduces intracellular signals generated by the Toll-like or IL-1 receptor (TLR or IL-1R) superfamily [14], [15]. Whereas TLRs are critical for detecting molecular patterns associated with microbial pathogens [16]–[20], IL-1 receptor (IL-1R1) is important for amplifying inflammatory responses triggered by microbial pathogens [21]. After ligand binding, dimeric MyD88 is recruited to the membrane-receptor complexes through the interaction of its C-terminal Toll-interleukin receptor (TIR) domain with an analogous domain in the IL-1R or TLR receptors [16]. The N-terminal death domain of MyD88 recruits the death domain-containing IL-1R-associated kinases (IRAKs) [14]. Activation of IRAK leads to a downstream signaling cascade that activates NF-kB, p38 mitogen-activated protein kinase (MAPK), and other factors, ultimately inducing production of inflammatory cytokines. Although TIR domain-linked interaction is important for MyD88-mediated signaling, MyD88 also interacts with interferon gamma receptor 1 (IFNGR1), which lacks a TIR domain [22]. This latter interaction enhances expression of pro-inflammatory cytokines in response to IFN-γ stimulation. Similar interactions have been detected between MyD88 and other proteins lacking TIR and death domains such as Bruton's tyrosine kinase [23], phosphatidyl-inositol-3-OH kinase [24], and interferon regulatory factor 7 (IRF7) [25]. These reports indicate that MyD88 can associate with signaling proteins by means other than homophilic TIR or death-domain interactions. We also found that MyD88^−/−^ mice were resistant to SE intoxication and had impaired pro-inflammatory cytokine responses [26], [27]. These results lead us to suggest that MHC class II molecules, which serve as signal-transducing receptors [28]–[30], may also utilize the cytosolic adaptor protein MyD88 for implementation of downstream signaling. We investigated the role of MyD88 signaling following MHC class II recognition of ligands such as SEB or SEA. In this study, we present direct evidence that engagement of MHC class II by SEB, SEA, or by anti-MHC class II antibodies in human primary monocytes and B cells, results in increased MyD88 signaling, NF-kB activation and cytokine responses.

## Materials and Methods

### Reagents

Staphylococcal enterotoxin B (SEB) and Staphylococcal enterotoxin A (SEA) were purchased from Porton Down, Inc. (Salisbury, UK), and was prepared under GMP condition, endotoxin free and stored at −50°C. *Escherichia coli* LPS (055:B5) was purchased from Difco laboratories (Detroit, MI.). Pooled human AB sera were obtained from Pel-Freez (Brown Deer, WI). A cytometric bead array kit for cytokines was purchased from BD Biosciences Pharmingen (San Diego, CA). RNA-extracting reagent Tri-Reagent was obtained from Molecular Research Center, Inc. (Cincinnati, OH), and Maloney murine leukemia virus reverse transcriptase was purchased from Perkin Elmer (Waltham, MA). Mouse anti-human CD14 and CD3 mAbs conjugated with magnetic beads were obtained from Miltenyi Biotech Inc. (Auburn, CA). The FITC-conjugated mAbs anti-CD14, anti-CD3, and Ig isotype control antibodies were purchased from BD Biosciences (San Jose, CA). Primary anti-MyD88 antibody was obtained from AnaSpec, Inc. (San Jose, CA) and Alexis Biochemicals (San Diego, CA). Anti- β-actin antibody was purchased from Imgenex (San Diego, CA) and Cell Signaling Technology (Danvers, MA). The mAb LB3.1 (anti-HLA-DRα) has been previously described [31] and was produced in ascites fluid. Purified OKT3 mAb was obtained from e-Bioscience (San Diego, CA). The mouse anti-human HLA-class II DP-DQ-DR mAb (MCA477), which recognizes HLA class II β chain of human MHC antigen, was obtained from Serotec, Ltd. (Oxford, UK). Goat anti-rabbit IgG (PE-labeled) was purchased from Pierce (Rockford, IL). Monoclonal anti-glyceraldehyde -3-phospahate dehydrogenase (GAPDH)-conjugated to horseradish peroxidase was purchased from Sigma Chemical Co. (St. Louis, MO). Maloney murine leukemia virus reverse transcriptase was purchased from Perkin Elmer (Waltham, MA). Agarose-bound anti-human MyD88 antibody was purchased from Santa Cruz Biotechnology (San Diego, CA). Anti-phosphotyrosine antibody and anti-IRAK-1 antibody was purchased from Cell Signaling Technology (Danvers, MA). Cell permeabilizing buffer was purchased from BD Biosciences Pharmingen (San Diego, CA). MHC class II antigen negative T2 cells (B cell line) were obtained from ATCC (Manasass, VA, USA). CpG-NT 10103 type B CpG oligonucleotide specifically optimized for vaccine applications was kindly provided by Chad Roy (USAMRIID) and has been described elsewhere [32]. Anti-TRAF6 antibody was purchased from Epitomics, Inc. (Burlingame, CA) and anti-IRAK4 antibody was purchased from MBL International (Woburn, MA).

### Human cell isolation from blood and culture

Peripheral blood mononuclear cells were obtained from healthy donors with written consents, in accordance with guidelines of the human use committee (HUC) and institutional (USAMRIID) review board-approved research donor protocol FY 05-05. Mononuclear cells were isolated by standard density gradient centrifugation with Ficoll-Hypaque (GE Healthcare Biosciences AB, Uppsala, Sweden), harvested from the interface, washed, and suspended in RPMI 1640 medium. Monocytes (CD14^+^) were purified as previously described [33]. Briefly, total mononuclear cells were suspended (10^7^cells/80 µl) in cold PBS supplemented with 2 mM EDTA and 0.5% bovine serum albumin (BSA) (Fraction V; Sigma Chemical Co., St. Louis, MO). Paramagnetic beads coated with anti-CD3 mAbs (Miltenyi Biotech) were mixed with the mononuclear cells (10^7^cells/20 µl). The antibody-bound cells were incubated for 15 min (4°C), washed, and passed through a type LS or RS iron-fiber column placed within a strong magnetic field (Miltenyi Biotech). CD3^+^ T-cells bound to the column were eluted with buffer after withdrawal from the magnetic field. Monocytes were isolated from cells not bound to the anti-CD3 column, by repeating the process above with anti-CD14 mAb-coated beads. Cell purities were >98% as determined by flow cytometry analyses of isolated mononuclear cell subsets used in all reported experiments.

### Cytokine assays

Cell cultures were incubated (37°C, 5% CO_2_) for 16 h. Supernatants were collected, and cells harvested for measuring transcriptional activation of the MyD88 gene and intracellular expression of MyD88 protein. Cytokines in culture supernatants were measured by a cytometric bead array kit (BD Biosciences Pharmingen) using capture beads coated with antibodies specific for cytokines. Flow cytometry analysis was done according to the manufacturer's method, as described elsewhere [32].

### NF-kB assays

We used ELISA-based chemiluminescence Trans AM Chemi Kit (Active Motif, Carlsbad, CA) and antibodies to detect NF-kB recognition of epitope on p50 and p65, according to the manufacturer's protocol. Assays were performed in triplicate to measure NF-kB by using equal amounts of protein from the cell extracts.

### RT-PCR

Total RNA was extracted from untreated and treated human monocytes, using Tri-Reagent (Molecular Research Center Inc,). The mRNA was reverse transcribed into cDNA with Maloney murine leukemia virus reverse transcriptase, according to the manufacturer's instructions. Amplification was performed with gene-specific primers, MyD88 (Forward 5′-CACTCGCAGTTTGTTGGATG-3′; Reverse 5′-CGCAGGATACTGGGAAAGTC-3′), and β-actin (Forward 5′- TCCTGTGGCATCCACGAAACT-3′; Reverse 5′-GAAGCATTTGCGGTGGACGAT-3′), TNF-α (Forward 5′- CGG GAC GTG GAG CTG GCC GAG GAG-3′; Reverse 5′-CAC CAG CTG GTT ATC TCT CAG CTC-3′); IL-1β (Forward 5′- AAA CAG ATG AAG TGC TCC TTC GAG G-3′; Reverse 5′- TGG AGA ACA CCA CTT GTT GCT CCA-3′). Human sIL-1RACP (Forward 5′-GATGGATTCTCGCAATGAGG-3′; Reverse 5′-ACTATGGGTTAGATGCGTC-3′);

Human mIL-1RACP (Forward 5′-GATGGATTCTCGCAATGAGG-3′;

Reverse 5′-TGAGAATCACCACTAGCAGG-3′); Human HLA-DR (Forward 5′-GCTATCAAAGAAGAACATGTG-3′; Reverse 5′-GAGCGCTTTGTCATATTTCCAG-3′). PCR products were separated by electrophoresis on a 1.5% agarose gel, stained with ethidium bromide, and photographed as previously described [34].

### Real-time PCR analysis

Semi-quantitative real time PCR was performed using cDNA collected as described above. Amplification was performed using 4 µl of a 1 µM final concentration of gene specific primers as described before, 10 µl of Power Sybr Green PCR master mix, and 6 µl cDNA according to the manufacturer's instructions. Finally, the 20 µl PCR reaction was amplified using a 7900HT Fast Real Time PCR System. The final normalized results were calculated by dividing the relative transcript levels of the test genes by the relative amount of the β-actin RNA.

### Western blot analysis

Human monocytes (CD14^+^), B cells (CD19^+^) or U937 cells were cultured in a 24-well plate at 2 −4×10^6^ cells/ml. These cells rested for 2 hrs in a 37°C incubator. Cells were treated with LPS at 1 µg/ml, SEB at 200 ng/ml, SEA at 200 ng/ml or left untreated for the indicated times. Cells were collected into fresh 1.5 ml centrifuge tubes and chilled on ice for 5 minutes before centrifuging. Membrane and cytoplasm separation was done by suspending the pellets in 50 µl of lysis buffer (Active Motif) in the presence of DTT, protease inhibitors and phosphatase inhibitors and incubated on ice for 30–60 minutes. Membrane fraction was collected by centrifuging the lysates at 14000×g for 20 minutes. Supernatant contained the cytoplasmic fraction and pellet contained membrane fraction. Samples containing 10 µg of total cytoplasmic proteins were separated by gel electrophoresis and transferred to nitrocellulose membranes. Membranes were blocked overnight in Tris-buffered saline containing 0.1% Tween 20 and 3% BSA at 4°C. Blots were extensively washed and probed with anti-MyD88 polyclonal antibody followed by HRP-conjugated secondary Ab. Blots were washed extensively and developed with chemiluminescent substrate in the presence of hydrogen peroxide using Immun-Star WesternC Chemiluminescent Kit (BioRad). An imaging system VersaDoc Model 4000 (BioRad) was used to capture the image.

### Co-immunoprecipitations

Monocytes were cultured in a 24-well plate at 2−4×10^6^ cells/ml of media and rested for 2 hrs in a 37°C incubator. Cells were treated with SEB at 200 ng/ml or left untreated for the indicated times. Cells were collected into fresh 1.5 ml centrifuge tubes and chilled on ice for 5 minutes before centrifuging. Wells were washed twice more with cold PBS. Cells were then resuspended in 1 ml of lysis buffer (50 mM Tris-HCl, 150 mM NaCl, 1% NP-40, pH 8.0), left on ice for 40 to 60 minutes and passed twice through a 26-gauge syringe. After lysing, samples were rotated overnight at 4°C in the presence of agarose-bound anti-human MyD88 Ab (Santa Cruz Biotechnology). After washing three times in cold PBS, the agarose pellets were resuspended in no more than 40 µl of loading buffer (5% β-mercaptoethanol) and boiled for 5 min. 20 µl per sample were separated by electrophoresis, transferred to nitrocellulose membranes and blocked overnight at 4°C and probed with a mouse anti-phosphotyrosine antibody. Blots were washed extensively and developed with chemiluminescent substrate in the presence of hydrogen peroxide using Immun-Star WesternC Chemiluminescent Kit (BioRad). An imaging system VersaDoc Model 4000 (BioRad) was used to capture the image.

### Intracellular staining and confocal microscopy

Freshly isolated monocytes (CD14^+^) adhered to sterile culture slides (Corning Glass, Corning, NY) were incubated with either SEA, SEB (200 ng/ml), mAb to anti-DP-DQ-DR, LB3.1, or OKT3 in RPMI 1640 medium. B cells or T2 cells were incubated with either CpG (10 µg/ml) or LPS 1 µg/ml in eppendorf tubes. Cells were gently washed with PBS (pH 7.4, 37°C) and then fixed with 1% paraformaldehyde (Tousimis Research, Rockville, MD) plus 0.1% glutaraldehyde (Sigma-Aldrich) in PBS (5 min, 20°C). The fixed cells were washed with PBS containing 0.5% bovine serum albumin (BSA) and incubated (30 min, 20°C) in a permeabilization solution (Becton Dickinson). Cells were then washed and incubated (15 min, 20°C) with PBS containing 5% BSA to block non-specific antibody binding. Cells were incubated with primary HLA-DR and MyD88 antibody for 1 h, washed in PBS, containing 0.5% BSA, then incubated with Alexa 488-conjugated goat anti-mouse secondary antibody and Alexa 568-conjugated goat anti-rabbit secondary antibody. Cells were counterstained with DAPI to detect cell nuclei. Labeled B and T2 cells were mounted on glass slides using fluoromount and covered with glass coverslips. Labeled cells were imaged using a BioRad (Hemel Hempstead, UK) Radiance 2000 MP laser scanning confocal system connected to a Nikon (Melville, NY) TE 300 inverted microscope. Green and red fluorescence were visualized using 488-nm and 568 nm wavelength excitation respectively from a krypton/argon laser, and DAPI images were collected using a two-photon excitation from a Ti/sapphire laser tuned to 800 nm. Images were collected using BioRad LaserSharp software. Images taken for the purpose of comparing control antibody-treated to MHC ligand-stimulated cells were collected using identical settings, and any subsequent processing of the images was likewise performed identically for the two conditions.

### Flow cytometry

To examine cell-surface expression of proteins on purified monocytes, cells were incubated (20 min, 4°C) with FcR-blocking reagent (Miltenyi Biotech) and washed twice with Hank's balanced salt solution (HBSS) containing 0.1% BSA. Cells were then incubated with FITC-labeled anti-CD14, anti-CD3 mAb, or isotype-matched control antibody. Unbound antibody was removed by washing the cells with HBSS (4°C) and centrifugation. After two additional washes, the labeled cells were fixed with 1% paraformaldehyde in PBS, and the cell-associated immunofluorescence was measured by flow cytometry (FACSCaliber, Becton Dickinson, San Jose, CA). Intracellular expression of MyD88 protein in CD14^+^ monocytes was examined using a modified method similar to intracellular staining of phosphorylated proteins in lymphocytes (BD Biosciences). Briefly, after treatment either with SEA, SEB, mAbs, or LPS, CD14^+^ monocytes were pelleted, washed once with PBS, and immediately fixed by mixing one volume of cells to 20 volumes of 1× Phosphoflow Lyse/Fix buffer (BD Pharmingen, San Diego, CA). Cells were incubated (10 min, 37°C), pelleted and permeabilized with PhosFlow Perm Buffer (III) (BD Pharmingen) for 30 min on ice, washed twice with BD Pharmingen TM stain buffer and pelleted by centrifugation (300× g) for 5 min. Cells were suspended (10^7^/ml) in BD Pharmingen stain buffer. BD FcR-block antibody (0.06 µg) was added for each 10^7^ cells, which were incubated on ice for 15 min. Cells were labeled with primary anti-MyD88 antibody (rabbit polyclonal) or isotype-matched control antibody followed by secondary goat anti-rabbit 568 (Molecular Probe). The labeled cells were washed, suspended in stain buffer and MyD88 expression was measured by flow cytometry.

### Statistical analysis

The SAS program version 9.2 (Cary, North Carolina) was used for statistical analysis. Repeated measures ANOVA were used for statistical analysis and pair wise comparisons of untreated vs treatment were used for real time PCR assays. Statistical significant differences between groups and control were determined by Student's T- test. Significant differences are indicated by asterisks and p values reported in figure legends.

## Results

### MyD88, IL-1R1, and TNF-α are up regulated in monocytes by engagement of HLA class II molecules

**Figure 1:**
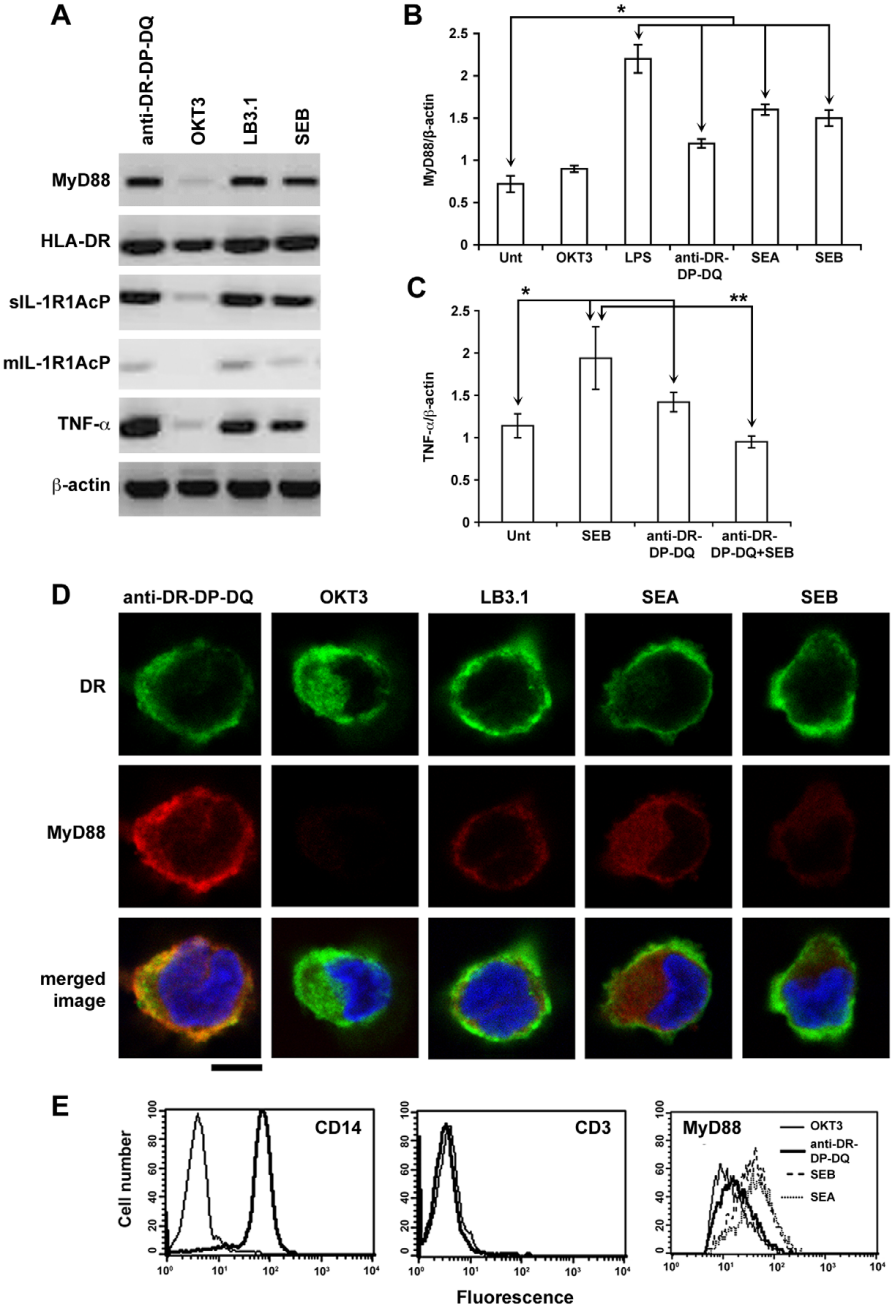
Ligand engagement of HLA class II molecules up regulates MyD88, IL-1R1, and TNF-α in CD14^+^ human monocytes treated with SEB, SEA, or mAb directed against MHC-class II molecules. (A) Transcriptional activation of MyD88, IL-1R1AcP and TNF-α in HLA-DR positive primary monocytes treated with 200 ng SEB/ml (optimum dose), mAbs (10 µg/ml, optimum dose) directed against MHC-class II molecule (anti-DR-DP-DQ or LB3.1) or unrelated control antibody OKT3 was examined by semi-quantitative RT-PCR. Data shown is one of 3 similar experiments; (B) Agonists binding to TLR4 or HLA class II molecules on CD14^+^ monocytes induced transcriptional up regulation of MyD88. Real time RT-PCR was used to determine relative expression of MyD88 normalized to the expression of β-actin. Expression levels are determined as means +/− SD. Data presented as one of 3 similar experiments. Significance compared to untreated control (*) was assigned as P≤0.0001. (C) MHC class II molecule dependence of SEB- induced TNF- α gene expression. Pretreatment of monocytes in ice with anti-DR-DP-DQ at optimum dose (10 µg/ml) followed by SEB stimulation resulted in reduced TNF- α gene expression. Transcriptional activation of TNF- α expression normalized to the expression of β-actin. Expression levels of TNF- α are expressed as means +/− SD. Significance was assigned (*) or (**) as P values ≤0.003 comparing untreated vs treatment groups with anti-DR-DP-DQ, SEB or SEB vs anti-DR-DP-DQ+SEB respectively. (D) Confocal images show expression of HLA-DR (green) and intracellular MyD88 (red) proteins in CD14^+^ monocytes treated with HLA class II- ligands or control antibody OKT3 for 16 h; Scale bar  = 5 µm; (E) intracellular expression of MyD88 protein in activated monocytes. Primary monocytes (CD14^+^, CD3^-^) were activated as described earlier, permeabilized and labeled with primary MyD88 antibody followed by PE-labeled secondary antibody and analyzed by flow cytometry. Histogram represents a MHC class II ligand-induced increase in expression of MyD88 protein compared to non-MHC class II ligand (OKT3).

MHC class II molecules on APCs act as signaling receptors for ligands such as SEB, SEA, or mAbs directed against MHC class II molecules. The binding of these specific ligands to MHC class II molecules induces pro-inflammatory responses including release of TNF-α and IL-1β. In the event of superantigen induced toxicity, it is known that SE binds to MHC Class II molecules on APCs and Vβ-containing T cell receptors, activating APCs and T cells and inducing pro-inflammatory cytokine production. We explored the possibility that induction of TNF-α and IL-1β by direct MHC class II- binding of SE in monocytes might be due to MyD88 activation. We treated human primary CD14^+^ monocytes (98% pure) with SEB, LB3.1 (mAb that recognizes an epitope on DR-α of HLA molecules), or anti-DR-DQ-DP (mAb directed against the DR-βchain of the HLA-DR molecule which also recognizes epitopes on HLA-DP and -DQ) for 2 h. MyD88, interleukin-1 receptor accessory protein (IL-1R1AcP), and TNF-α mRNA were all substantially induced at 2 h (Figure 1A) as detected by RT-PCR. Similar data were collected at 16 h (data not shown). In contrast, the control mAb (OKT3) did not activate the cells (Figure 1A). The IL-1R1AcP acts as a co-receptor for IL-1R1 and is an indispensable molecule in the IL-1R1 signal transduction complex [34]. Induction of the membrane-bound form of IL-1R1AcP by SEB (Figure 1A) is particularly worthy of note because it is an essential component of the trimeric IL-1/IL-1 receptor/mIL-1RAcP complex necessary for downstream MyD88 signaling [35]. To confirm the RT-PCR results, we used real time PCR for accurate and reproducible measurement of MyD88 mRNA levels in monocytes stimulated with SEA, SEB, LPS, OKT3 and anti-DR-DP-DQ. The real time PCR analysis confirmed that, similar to LPS activation, monocytes treated with MHC class II ligands such as SEA, SEB, and anti-DR-DP-DQ significantly increased transcription of MyD88 compared to unstimulated monocytes or control antibody (OKT3) (Figure 1B). Importantly, prior treatment of monocytes with anti-DR-DP-DQ antibody in ice followed by SEB stimulation significantly reduced transcription of MyD88 (data not shown) as well as TNF-α gene (Figure 1C) as detected by real time PCR indicating that SEB acts through HLA and not other surface components. In addition to transcriptional activation of MyD88 in primary monocytes, confocal microscopy and flow cytometry analysis indicated a substantial increase in intracellular expression of MyD88 protein levels following treatment with SEB, SEA, or LB3.1 or anti-DR-DP-DQ compared to the control antibody OKT3 (Figure 1D and E). Although direct interaction of HLA-DR and MyD88 cannot be determined from confocal images, the partial overlap of fluorescence observed in the images, particularly with anti-DR-DP-DQ mAb-treated cells, is consistent with this possibility. Results shown in Figure 1D indicate HLA-DR ligands initiated up regulation of MyD88 protein above the levels of the controls. Flow cytometry analysis using an isotype matched control antibody did not show up regulation of MyD88 (data not shown). In Figure 1E the FACS showed along with anti-DR-DP-DQ, SEA or SEB treatment increased up regulation of MyD88. The effect was greater with SEA or SEB. Collectively, results shown in Figure 1A-E suggest that engagement of MHC class II molecules either by SEA, SEB or anti-MHC class II antibody up regulated MyD88. It is important to note that these data were from primary monocytes isolated from different donors. The level of up regulation appeared to have some normal donor –to- donor variation. In addition to RT-PCR and real time PCR, confocal and FACS analysis were used to confirm these results at a single cell level (Figure 1D) as well as multiple cell level (Figure 1E). The results in Figure 1 consistently suggest that engagement of MHC class II molecules by SEA, SEB or anti-MHC class II antibody up regulated MyD88.

### SEB stimulation of monocytes along with up regulation of MyD88, activates NF-kB and cytokines

**Figure 2:**
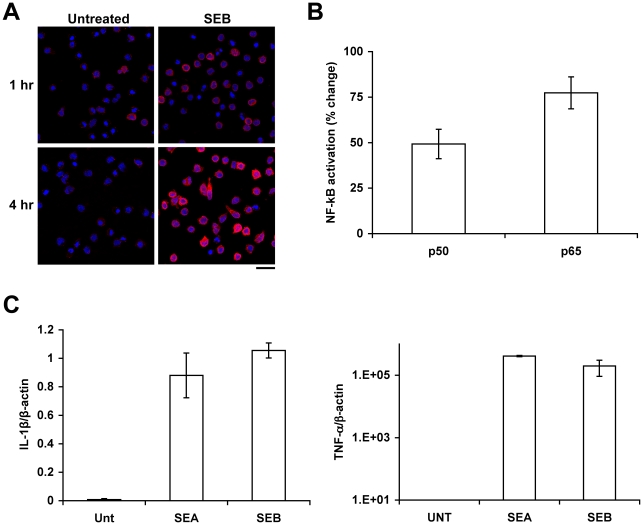
SEB stimulation of monocytes activates MyD88, NF-kB, TNF-α, and IL-1β. SEB engagement of HLA-class II on human monocytes induced intracellular up regulation of MyD88, TNF-α, and IL-1β compared to untreated control and additionally activated NF-kB. (A) Confocal images show intracellular up regulation of MyD88 protein in primary monocytes stimulated with SEB for 1 h compared to untreated monocytes; Scale bar = 10 µm. (B) Activation of NF-kB in primary monocytes treated with SEB. Data are presented in the figure as percentage increase over the untreated control and represent one of three experiments using separate donors. Significance was assigned as follows; p50 (p = 0.009) and p65 (p = 0.004). (C) Transcriptional activation of IL-1β, and TNF-α mRNA was measured by real time RT-PCR in isolated primary monocytes after stimulation of total mononuclear cells with SEA or SEB.

To further validate our observation that intracellular MyD88 synthesis is consistently up regulated in a vast majority of the monocytes as a consequence of SEB stimulation, a more detailed analyses of the effects of SEB binding was examined by comparing a large number of untreated vs SEB treated monocytes following 1 and 4 h of treatment using microscopy. Results shown in Figure 2A indicate that, compare to untreated cells, MyD88 was strongly up regulated 1 and 4 h after SEB stimulation in monocytes. Activation of NF-kB p50 and p65 was also seen in monocytes after SEB stimulation (Figure 2B). Next we examined whether SEB- induced up- regulation of MyD88 in monocytes is physiologically relevant and consistent in the presence of T cells. For this, we examined up regulation of IL-1β and TNF-α in purified monocytes that were separated from the total mononuclear cell population stimulated with SEB and from total unstimulated mononuclear cells (Unt). Our results showed that, consistent with up regulation of MyD88 protein, IL-1β and TNF-α expression were up regulated either with SEB as well as SEA treatment compared to untreated controls (Figure 2C). Taken together, these results suggest that MHC class II based recognition of SEA or SEB in monocytes alone or within a total mononuclear cell population induces MyD88 up regulation and downstream signaling via NF-kB, potentially enabling pro-inflammatory cytokine response. MyD88 up regulation was also observed in T cells (data not shown).

### *De novo* synthesis, homodimerization and phosphorylation of MyD88 after SEB stimulation of monocytes

**Figure 3:**
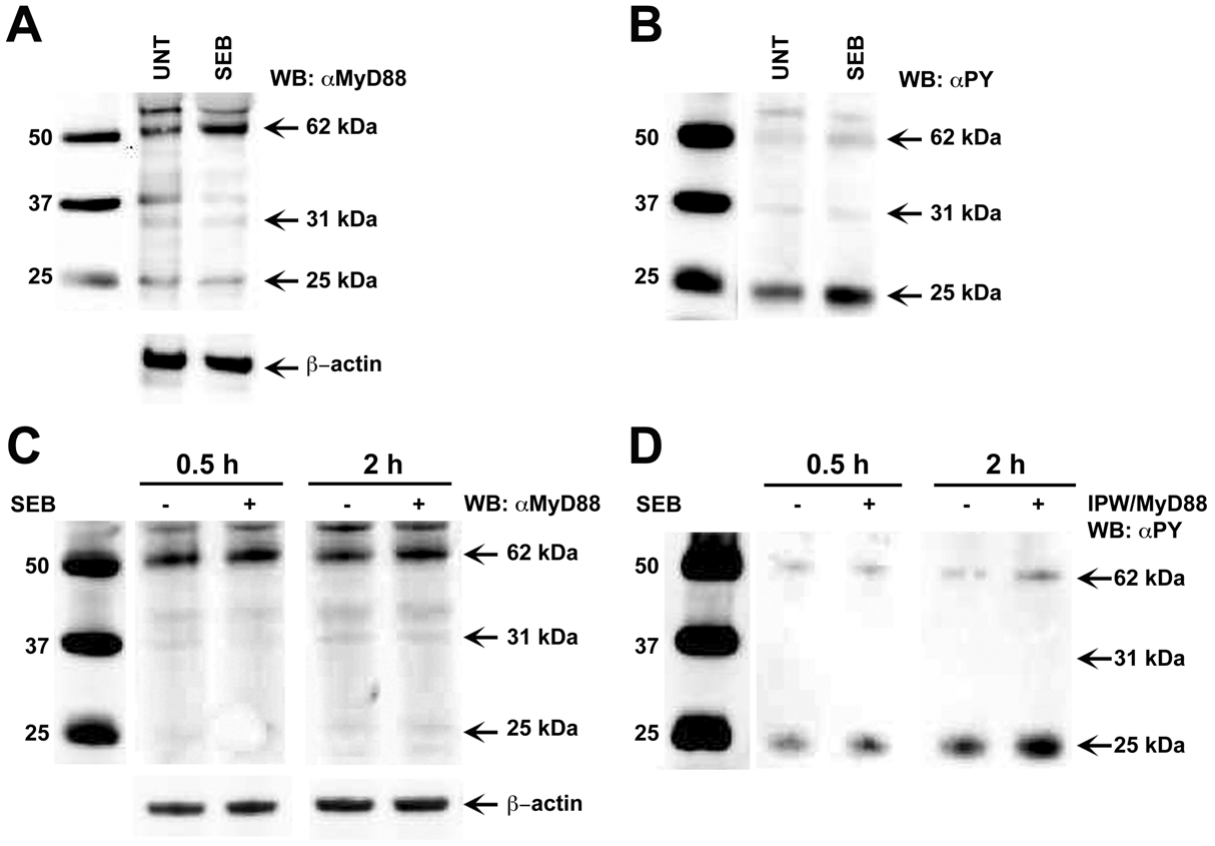
SEB stimulation induced *de novo* synthesis and phosphorylation of MyD88. CD14^+^ cells were treated with SEB for different times or left untreated as indicated. Cells were lysed, membrane fraction and cytoplasm fraction were isolated by centrifugation. Cytoplasmic fractions were run by electrophoresis and blotted using an anti-human MyD88 antibody. (A) Up regulation of MyD88 (62 kDa) dimer and 31 kDa monomer after SEB stimulation. Monocytes were treated with SEB (200 ng/ml) for 1 h or left untreated. (B) Phosphotyrosine antibody recognized MyD88 (25 kDa) band in cytoplasmic extracts of monocytes stimulated with SEB. (C) MyD88 protein up regulation in monocytes after SEB stimulation. Monocytes (CD14^+^) were treated with SEB (200 ng/ml) for 30 min or 2 h or left untreated. After treatments, cytoplasmic fractions were isolated, separated by electrophoresis and probed with anti-MyD88 or anti-β-actin antibody. (D) SEB stimulation of monocytes induced phosphorylation of MyD88. Part of the cytoplasmic fractions isolated after SEB stimulation as described in (C) was immune-precipitated with an agarose-bound anti-human MyD88, separated by electrophoresis and blotted against an anti-phosphotyrosine antibody (α-PY).

Recruitment of MyD88 dimer to the receptor-membrane complex is a requirement for MyD88- mediated signaling and activation of the down-stream kinases IRAK1 and IRAK4 [36]. MyD88 exists as a homodimer with a molecular mass of approx. 62–66 kDa but could also be detected as a monomer with a molecular mass of 31–33 kDa in SDS-PAGE [37]. To further substantiate that SEB binding to MHC class II molecules activates MyD88, we stimulated primary monocytes with SEB. Western blot analysis showed up regulation of MyD88 in SEB treated monocytes compared to untreated cells, consistent with confocal microscopy and flow cytometry results. An increase in MyD88, as two bands of an approximate molecular mass of 62–66 kDa (homodimer) and 31–33 kDa (monomer) was observed in activated cells while β- actin levels remained the same (Figure 3A). Protein expression bands in the Western blots were quantified band intensities and compared untreated vs SEB activated monocytes using 10 µg and 20 µg protein of cytoplasmic fractions (Figure S1). These results are consistent with an earlier report where *in vivo* MyD88 expression was observed as a dimer (62–66 kDa) as well as a monomer (31 kDa) using SDS-PAGE/Western analysis [37]. To our knowledge, this is the first report which suggests that primary monocytes after SEB stimulation up regulated MyD88, exhibited as a dimer as well as monomer. A similar observation was made in monocytes collected from multiple donors (Figure S2). However, the levels of up regulation of the dimer varied from donor to donor which is not unexpected. Furthermore, probing with phosphotyrosine antibody, a 25 kDa band became especially prominent (Figure 3B). However, this band was not prominent in cytosolic fractions in Western Blot analysis when probed with MyD88 antibody raised to the C-terminal 233–248 aa region, suggesting that the 25 kDa protein may likely be a degraded MyD88 (Figure 3A). To further confirm phosphorylation of MyD88 after SEB stimulation, we performed immunoprecipitation of whole cell lysates by anti-MyD88 antibody raised against full length MyD88 protein (1–296 aa), and probed with phosphotyrosine antibody in Western blot analysis. The 62 kDa protein was recognized when probed with anti-MyD88 antibody (Figure 3C). However, the 62 kDa protein was barely detectable with anti-phosphotyrosine antibody whereas the 25 kDa band showed very strongly in immunoprecipitated extracts (Figure 3D), suggesting that the 25 kDa band is possibly degraded after phosphorylation of MyD88. It has been previously reported that phosphorylation of signaling adaptor Mal is required for Mal to signal [38]. A very recent report indicated that in macrophage TLR signaling SyK interacted with and induced tyrosine phosphorylation of MyD88, which led to degradation of these adaptor molecules by the E3 ubiquitin ligase Cbl-b [39]. Also PYK2, a major cell adhesion-activated tyrosine kinase interacts with MyD88 and appeared to be tyrosine phosphorylated upon coexpression of PYK2 and regulates MyD88-mediated NF-kB activation in macrophages [40]. In this study, our results suggest that SEB stimulation of monocytes induced phosphorylation of MyD88. A strong 25 kDa band was detected after immunoprecipitation with anti- MyD88 antibody followed by probing with phosphotyrosine antibody which suggests that MyD88 undergo phosphorylation, polyubiquitination and degradation. Taken together, these results suggest that engagement of HLA molecules by SEB allowed *de novo* synthesis and phosphorylation of MyD88.

### Up regulation of IRAK4, IRAK1 and TRAF6 after SEB stimulation of primary monocytes and U937 cells

**Figure 4:**
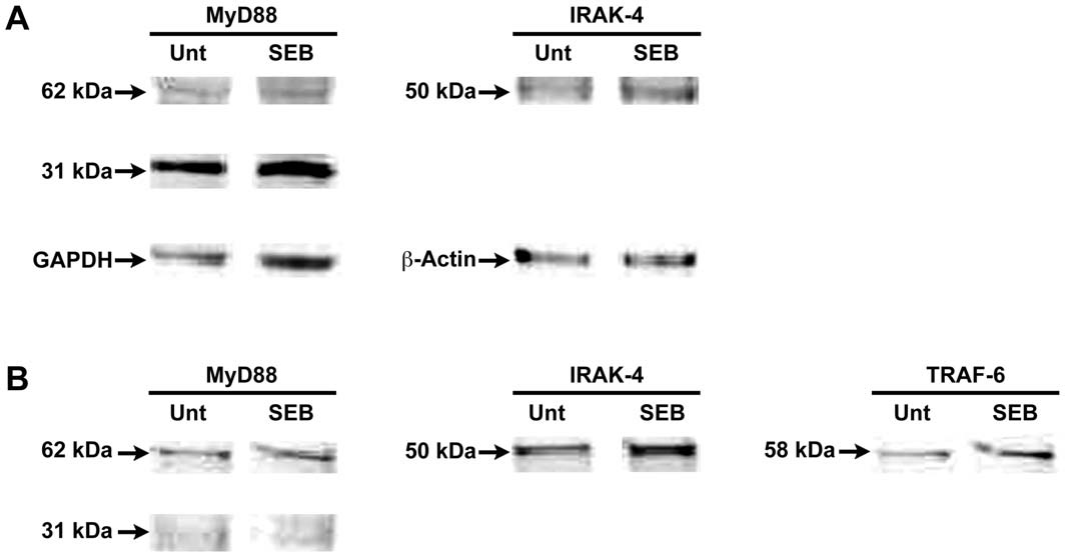
SEB stimulation induced up regulation of IRAK4 and TRAF6 in primary monocytes and U937 cells. Primary monocytes or human monocytic cell line U937 were treated with SEB or left untreated as indicated. Cells were lysed, membrane fraction and cytoplasm fraction were isolated by centrifugation. Cytoplasmic fractions were run by electrophoresis and blotted using an anti-human MyD88 antibody or anti-IRAK4, or anti-TRAF6 antibody. (A) Up regulation of MyD88 (62 kDa) dimer and 31 kDa monomer, TRAF6, IRAK4 after SEB stimulation in monocytes (B) Up regulation of MyD88 (62 kDa) dimer and 31 kDa monomer, TRAF6, IRAK4 after SEB stimulation in U937 cells.

The functional activation of MyD88-mediated pro-inflammatory signaling is enabled through engagement of the MyD88-downstream signaling components such as IRAK1, IRAK4 as well as TRAF6. To validate that SEB stimulation activated MyD88-mediated signaling, we treated primary monocytes and monocytic cell line U937 with SEB and examined IRAK4, IRAK1 and TRAF6 along with MyD88 in Western blot analysis. Results shown in Figure 4 indicate that in addition to MyD88, downstream signaling components such as IRAK4 and TRAF6 were also up regulated. We further confirmed these results in monocytes from different donors that showed up regulation of IRAK1 by striping the blot and rebloting with anti-MyD88 antibody and β-actin (Figure S3). Taken together, these results suggest that engagement of HLA-DR molecules with SEB enabled functional activation of MyD88 signaling in monocytic cells via activation of downstream signaling components.

### MHC class II-linked up regulation of MyD88 in primary B cells

**Figure 5:**
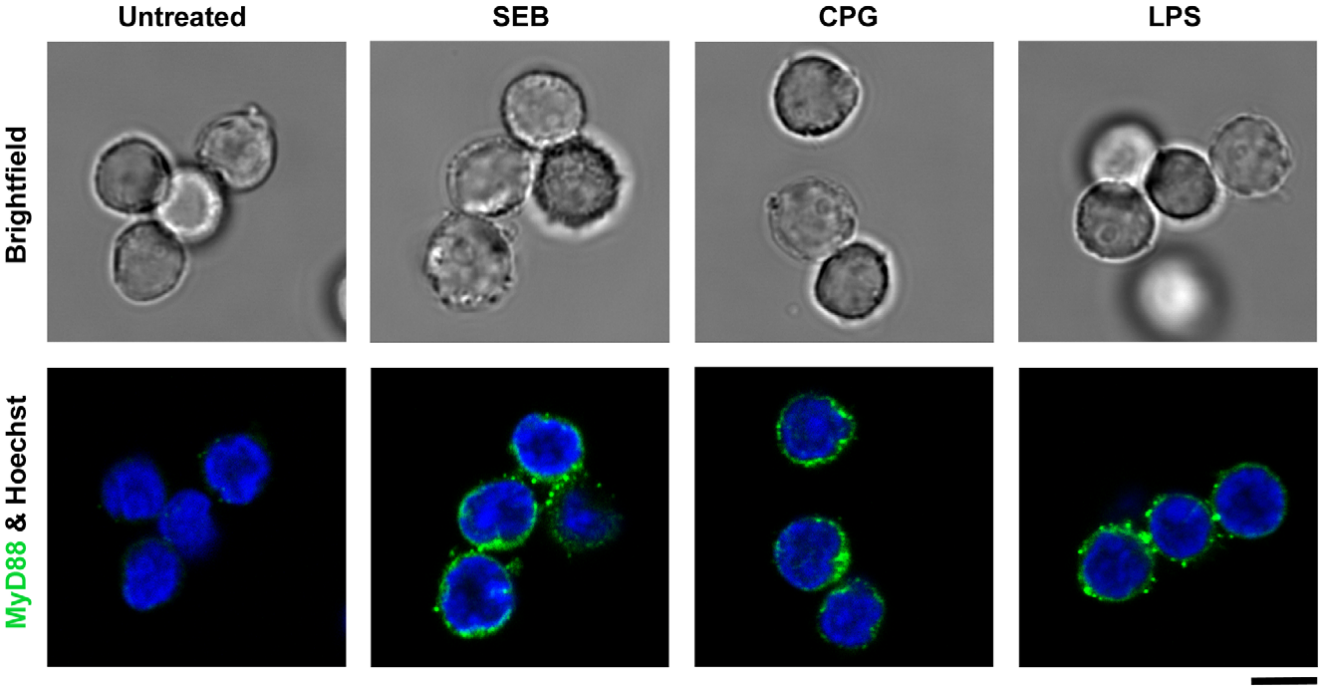
MHC class II-linked up regulation of MyD88 in primary B cells. Primary B cells (CD19^+^) were cultured in the presence of SEB (200 ng/ml), CpG (10 µg/ml), or LPS (1 µg/ml), fixed, permeabilized and labeled with anti-human MyD88 antibody. Confocal images show intracellular up regulation of MyD88 protein in primary monocytes stimulated with SEB, LPS or CpG compared to untreated cells; Scale bar  = 5 µm.

Ligation of MHC class II molecules in B cells can trigger intracellular signaling via the activation of tyrosine kinases and protein tyrosine kinase C [6], [8], [11], [12]. TLR engagement by LPS or CpG is also known to be involved in pro-inflammatory signaling via MyD88 in B cells. To characterize MHC class II- linked up regulation of MyD88 in cells other than monocytes, we examined MHC class II positive primary B cells (CD19^+^, MHC class II ^+^) after SEB stimulation. Confocal microscopy results showed that, compared to untreated cells, intracellular MyD88 was up regulated in primary B cells when stimulated with SEB as well as with LPS or CpG (Figure 5). These results suggest that similar to LPS or CpG, SEB up regulated MyD88 in B cells.

### B cells but not MHC class II- deficient B-cells, respond to SEB and up regulate MyD88

**Figure 6:**
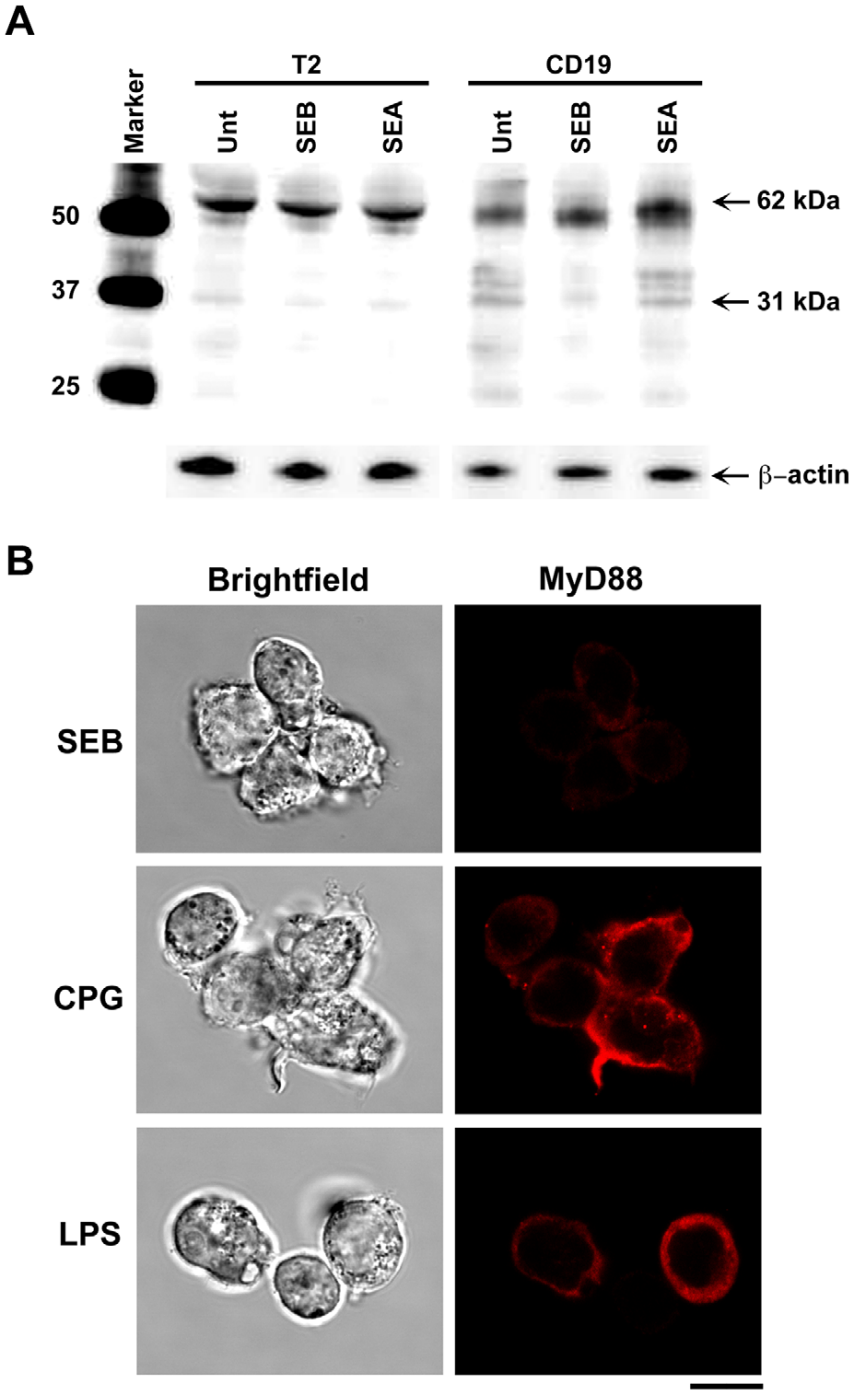
B cells but not MHC class II deficient T2 cells respond to SEB and activate MyD88. B cells or T2 cells were treated with SEA (200 ng/ml) or SEB (200 ng/ml) or kept untreated. Cells were lysed, membrane fraction and cytoplasm fraction were isolated by centrifugation. Cytoplasmic fractions were run by electrophoresis and blotted using an anti-human MyD88 antibody. (A) Detection of MyD88 after SEA and SEB stimulation in B cells or T2 cells. (B) Intracellular up regulation of MyD88 (red) in T2 cells after CpG and LPS stimulation; Scale bar  = 10 µm.

To confirm MHC class II-linked activation of MyD88 in B cells, we compared the effects of SEA or SEB stimulation on MyD88 levels in primary B cells and MHC class II negative T2 cells. Our results demonstrated that SEA or SEB stimulation of primary B cells up regulated MyD88 dimer whereas MHC class II negative T2 cells did not (Figure 6A). However, non-MHC class II-linked B cell ligands such as CpG or TLR-dependent LPS did activate MyD88 in T2 cells (Figure 6B). These results suggest that SEB induces up regulation of MyD88 dependent to MHC class II receptor in primary B cells.

## Discussion

In this study, our results show that engagement of HLA -class II molecules by either mAbs or the superantigens SEB and SEA up regulated MyD88 and increased expression of IL-1R1, IL-1β, and TNF-α. This MHC class II-linked up regulation of MyD88 after SEA or SEB stimulation and pro-inflammatory cytokine expression was observed in purified monocytes as well as in primary B cells, but not in MHC class II deficient B cells. Furthermore, MyD88 activation was associated with *de novo* synthesis and phosphorylation of MyD88. Importantly, our results also suggest that along with MyD88, SEB stimulation induced up regulation of other downstream signaling components such as IRAK4 and TRAF6. Typically, activation of TLR/IL-1R –MyD88-mediated signaling initiates with the recruitment of MyD88 to the receptor complexes. MyD88 primarily functions because its TIR domain heterodimerizes with the receptor and homodimerizes with another MyD88 molecule to recruit downstream signaling molecules. This includes the IRAK1-4 kinases via MyD88's death domain that ultimately results in activation of NF-kB and the expression of inflammatory cytokines such as TNF-α, IL-1β and IL-6.

However, a recent report indicated that IFN-γ stimulation of macrophages resulted in physical association of MyD88 with IFN-γR1, which naturally lacks the TIR domain [22]. Our recent results indicated that MyD88-mediated pro-inflammatory signaling is critical to SEA or SEB induced toxicity. SEs bind to MHC class II on APCs and to T- cell receptors, activating both cell types and inducing pro-inflammatory responses. In this study, our data suggest that MHC class II receptor-SE interaction up regulates MyD88, IRAK4, TRAF6, NF-kB and cytokine expression in monocytes.

Although the precise role of MHC class II molecules in signal transduction remains unclear, overwhelming evidence indicates that MHC class II molecules serve as signaling proteins in diverse antigen-presenting cells such as B cells, dendritic cells, and monocytes. HLA-DR-specific antibodies and SEB, which bind directly to MHC class II molecules, induce IL-1 production in human monocytes and myeloid cell lines [6], [7]. Thus, MHC class II molecules serve as signaling proteins in diverse antigen-presenting cells. Anti-MHC class II antibodies induce early biochemical signals, like a rise in cyclic AMP accompanied by nuclear translocation of protein kinase C in murine B cells [41]. Cross-linking of MHC class II antibodies induces the accumulation of inositol phosphates, increases intracellular calcium concentrations, and enhances tyrosine phophorylation in human B cells [9], [10]. Thus, ligands that engage MHC class II molecules trigger intracellular signaling in human monocytes, monocytic cell lines, and B cells through tyrosine phosphorylation of cellular proteins by activated protein kinase C and protein tyrosine kinases [7], [8]. However, besides the involvement of protein tyrosine kinase induction of inflammatory cytokines in response to SEB, other plausible mechanisms for intracellular signaling have not been described in the literature. Hence, SEA or SEB induced activation of MyD88 and the subsequent pro-inflammatory signaling has not been previously characterized. Ours is the first demonstration of MyD88 up regulation following MHC class II –SE interaction in monocytes and in B cells but not in MHC class II negative cells. A recent report [42] indicated that in B cells MyD88 is required for the production of natural antibodies, which is impaired in MyD88^B−/−^ chimeric mice due to a lack of B cell signaling via MyD88.

The pro-inflammatory cytokines TNF-α and IFN-γ use signaling pathways that involve Janus kinases and signal transducers and activators of transcription (STATs) [43]. However, MyD88 has no known function in these pathways. Earlier studies showed that both PTK and PKC play essential roles in HLA-class II molecule-mediated signal transduction induced by SEB and suggested the possibility that SEB induced IL-1β and TNF-α gene expression are differently regulated by distinct additional regulatory pathways [8], [44]. In this study, our results uncover a key process of MHC class II receptor signaling through recruitment of MyD88. Further, no increase of TNF-α by MHC class II positive cells from MyD88 knockout mice were observed after SEA stimulation suggests the existence of a MHC class II-linked MyD88-mediated signaling mechanism [26]. These data provide a novel concept that signaling via the MyD88 pathway is not limited to the TLR/IL-1R, but rather is generally shared by other receptors such as MHC receptors. Previous reports indicated that intra-cytoplasmic domains of MHC class II molecules and lipid-rich microdomains play a key role in MHC class II-mediated signaling [45]. It was also reported that CD38 and HLA-DR are functionally and physically associated in lipid raft microdomains at the cell surface of monocytes. Tetraspanin CD9 molecules and lipid raft microdomains are necessary for HLA-DR and CD38 signaling events [46]. Although the underlying mechanism by which MHC class II recruits MyD88 needs further investigation, as the adaptor protein is known to integrate signals through TIR domain-TIR domain interactions [16]. Recently it was reported after LPS or peptidoglycan stimulation of macrophages required MyD88 for PYK2 induced NF-kB activation and MyD88 appeared to be tyrosine-phosphorylated [40]. To our knowledge, this report provides the first evidence that MHC class II molecules, which serve as signaling receptors despite the absence of a TIR domain, recruit cytosolic MyD88 for inflammation. Importantly, our data also demonstrates MyD88-mediated amplification of pro-inflammatory cytokines upon SEB binding to MHC class II molecules on monocytes, even in the absence of T-cells. In a related study, we also observed significant up regulation of intracellular MyD88 in CD11c positive cells and macrophages isolated from the spleens of mice inoculated with SEB [27]. Our study describes MHC class II linked activation of MyD88-mediated pro-inflammatory signaling among isolated monocytes, as well as in human mixed PBMC cultures stimulated with SEB or SEA. These results suggest that SEA or SEB binding to MHC class II molecules to CD14^+^ monocytes leads to MyD88 activation and pro-inflammatory signaling. The biological effect of SEB is thought to result in cross-linking of T-cell receptors and MHC class II in the absence of conventional antigen processing. It has also been reported that [47] MHC class II-peptide-specific T cells responded to ova peptide in a MyD88- dependent manner. MyD88^−/−^ mice failed to elicit Th1 cytokine response to ova peptide. Thus, a linkage of MHC class II and MyD88 in antibody [42] and T cell response is beginning to emerge.

MyD88 is a required component for promoting productive T cell-independent (TI) B cell receptor (BCR) signaling where Bruton's tyrosine kinase (BTK, a cytoplasmic kinase that lacks TIR domain but is crucial for BCR-mediated activation) participates in a TI antibody response [48]. The most striking result of this earlier study was that while the absence of BTK and MyD88 entirely abrogated *Borrelia hermsii* Ag-specific IgM antibody production, it remained relatively robust in the absence of BTK alone. Thus, without BTK and normal BCR signaling, *B. hermsii* is capable of rapidly activating Ag-specific B cells, presumably by stimulating other signaling pathways [48]. It is an intriguing possibility that the MHC class II –MyD88 mediated signaling described in our current study could also be involved in an alternative functional pathway for antibody responses. Although the current study did not investigate the role of proteins that are likely associated with MHC class II in the activation of MyD88–mediated signaling, their involvement nonetheless remains a distinct possibility. Further investigation of the MHC class II–MyD88 interaction will elucidate not only the molecular mechanism underlying MHC class II-MyD88-mediated activation of inflammatory responses, but also the *in vivo* significance.

## Supporting Information

Figure S1 **SEB stimulation up regulated MyD88 compared to untreated monocytes.** (A) Up regulation of MyD88 after SEB stimulation. (B) Quantification of MyD88 protein expression (62kDa) in cytoplasmic fractions (20 µg and 10 µg) from untreated and SEB treated monocytes compared to β-actin expression in Western blot analysis.
(TIF)

Figure S2 **MyD88 up-regulation after SEB stimulation of monocytes isolated from three independent donors.** Monocytes were treated with SEB (200 ng/ml) for 1h or left untreated. Cells were lysed, membrane fraction and cytoplasm fraction were isolated by centrifugation. Cytoplasmic fractions were run by electrophoresis and blotted using an anti-human MyD88 antibody and anti-GAPDH antibody was used as control.
(TIF)

Figure S3 **SEB stimulation up regulated IRAK1 and MyD88 as compared to untreated monocytes.** Monocytes were treated with SEB (200 ng/ml) for 1h or left untreated. Cells were lysed, membrane fraction and cytoplasm fraction were isolated by centrifugation. Cytoplasmic fractions were run by electrophoresis and blotted using an anti-IRAK1 antibody, blot was sequentially striped and reprobed with anti-human MyD88 antibody, and β-actin.
(TIF)
