# Supplementary figures and images for: Activation of MyD88 Signaling upon Staphylococcal Enterotoxin Binding to MHC Class II Molecules
Source: PLoS One. 2011 Jan 20;6(1):e15985. doi: 10.1371/journal.pone.0015985 (PMC3024394; doi:10.1371/journal.pone.0015985)

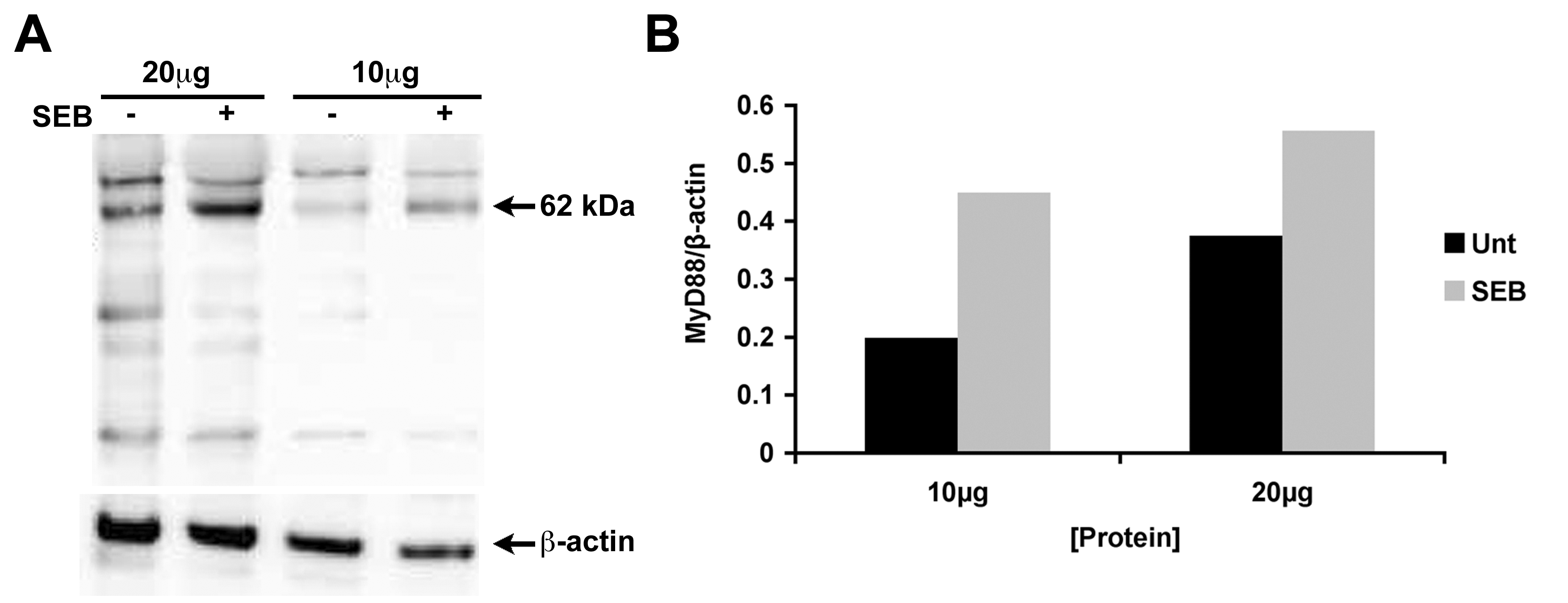

Supplement: Figure S1 — SEB stimulation up regulated MyD88 compared to untreated monocytes. (A) Up regulation of MyD88 after SEB stimulation. (B) Quantification of MyD88 protein expression (62kDa) in cytoplasmic fractions (20 µg and 10 µg) from untreated and SEB treated monocytes compared to β-actin expression in Western blot analysis. (TIF) [file pone.0015985.s001.tif]

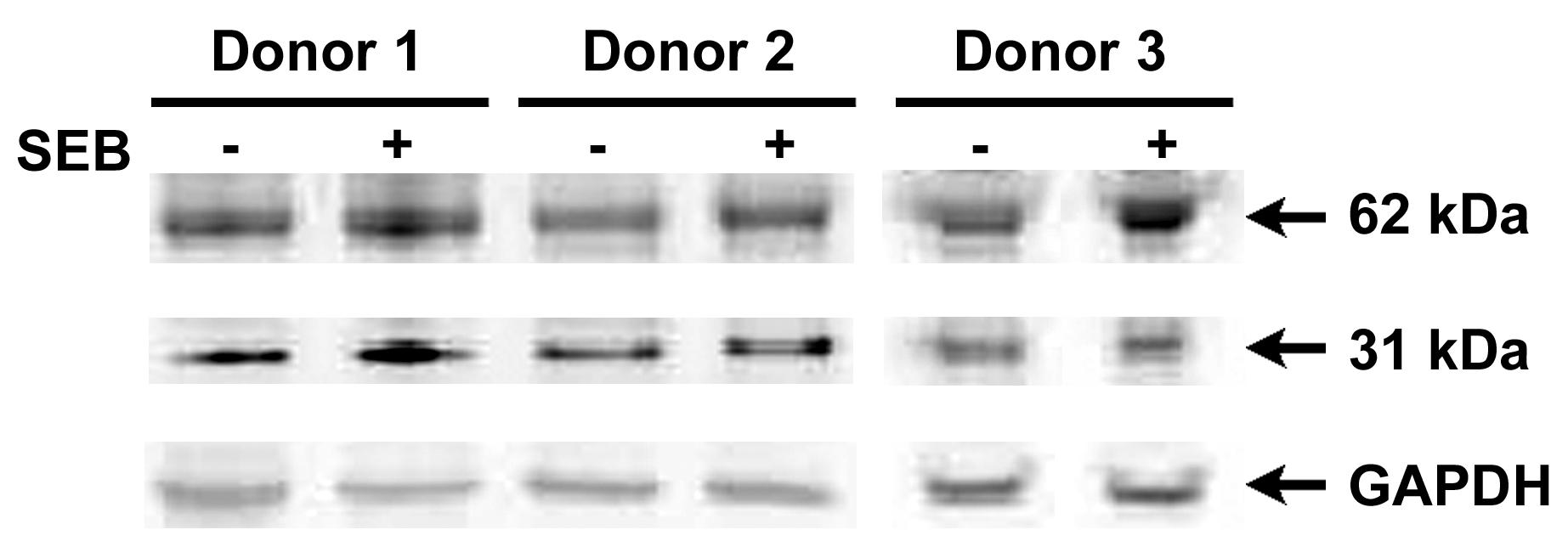

Supplement: Figure S2 — MyD88 up-regulation after SEB stimulation of monocytes isolated from three independent donors. Monocytes were treated with SEB (200 ng/ml) for 1h or left untreated. Cells were lysed, membrane fraction and cytoplasm fraction were isolated by centrifugation. Cytoplasmic fractions were run by electrophoresis and blotted using an anti-human MyD88 antibody and anti-GAPDH antibody was used as control. (TIF) [file pone.0015985.s002.tif]

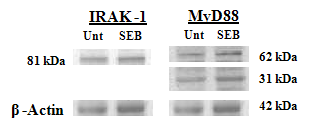

Supplement: Figure S3 — SEB stimulation up regulated IRAK1 and MyD88 as compared to untreated monocytes. Monocytes were treated with SEB (200 ng/ml) for 1h or left untreated. Cells were lysed, membrane fraction and cytoplasm fraction were isolated by centrifugation. Cytoplasmic fractions were run by electrophoresis and blotted using an anti-IRAK1 antibody, blot was sequentially striped and reprobed with anti-human MyD88 antibody, and β-actin. (TIF) [file pone.0015985.s003.tif]
